# Association between serum albumin-to-globulin ratio and subtypes of cerebral atherosclerotic stenosis in acute ischemic stroke

**DOI:** 10.3389/fneur.2025.1666940

**Published:** 2025-10-16

**Authors:** Yu-xin Fu, Jie Cao, Xiang Yin, Yue Lang, Teng-fei Su, Li Cui

**Affiliations:** Department of Neurology, The First Hospital of Jilin University, Changchun, China

**Keywords:** albumin-to-globulin ratio, acute ischemic stroke, cerebral atherosclerotic stenosis, posterior circulation stenosis, intracranial vascular stenosis

## Abstract

**Background:**

The albumin-to-globulin ratio (AGR) is a biomarker reflecting both nutritional status and inflammation, which has recently been implicated in the development of ischemic stroke. However, its potential association with the occurrence of cerebrovascular stenosis remains unclear. This study aimed to investigate the relationship between AGR and the incidence of cerebral atherosclerotic stenosis.

**Methods:**

Data from 766 adult patients with acute ischemic stroke (AIS) were included in this cross-sectional analysis. Binary logistic regression was used to evaluate the independent association between AGR and the risk of various cerebrovascular stenosis, including anterior circulation stenosis, posterior circulation stenosis, intracranial and extracranial stenosis. To explore the potential non-linear relationship between AGR and these outcomes, restricted cubic spline models were employed to further clarify these associations. Stratified analyses by body mass index (BMI), age, and sex were additionally conducted to explore the correlation between AGR and cerebral atherosclerotic stenosis under different conditions.

**Results:**

Patients with cerebral atherosclerotic stenosis had lower AGR levels than those without corresponding vascular stenosis. After adjusting for multiple covariates, AGR levels were negatively associated with the presence of stenosis in the posterior circulations (OR = 0.59, 95%CI = 0.38 ~ 0.90, *p* = 0.015) and intracranial stenosis (Q4: OR = 0.55, 95% CI = 0.34 ~ 0.89, *p* = 0.015). This association was essentially unaffected by BMI, age, or sex. Furthermore, a negative linear relationship was observed between AGR levels and the occurrence of posterior circulation stenosis (*p* for overall = 0.001, *p* for non-linear = 0.228) and intracranial vascular stenosis (*p* for overall <0.001, *p* for non-linear = 0.440).

**Conclusion:**

Higher AGR is associated with a reduced risk of multiple cerebral atherosclerotic stenosis. AGR levels are significantly associated with the presence of specific stenosis subtypes and could be hypothesized as a marker for risk stratification; this utility requires validation in prospective cohorts.

## Graphical abstract

**Figure:**
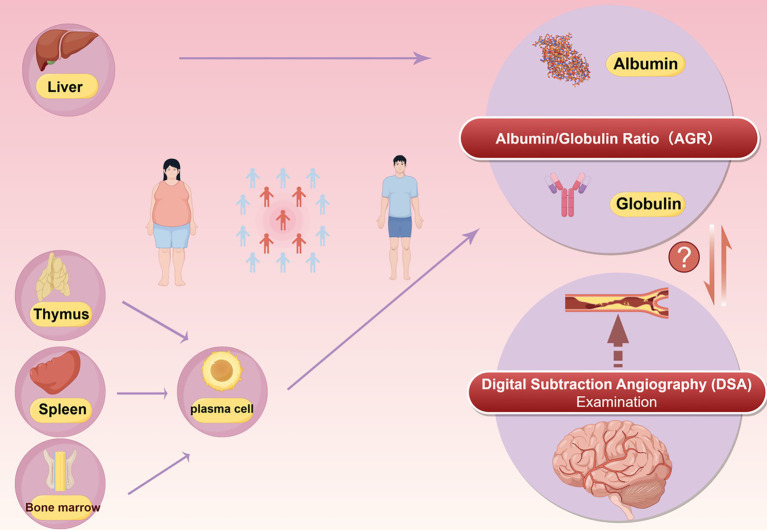
The association between serum albumin-to-globulin ratio and cerebral atherosclerotic stenosis in patients with acute ischemic stroke: a study stratified by body mass index, age, and sex.

## Background

Stroke represents a critical global health challenge, with ischemic stroke accounting for 65.3% of all stroke cases and ranking as the third leading cause of mortality worldwide (1). Between 1990 and 2021, inadequate management of modifiable risk factors—including obesity, elevated systolic blood pressure, and hyperglycemia—has further contributed to the rising incidence and socioeconomic burden of stroke (1). Therefore, the continuous development and implementation of effective and accessible strategies for early detection and prevention of stroke are of paramount importance. Ischemic stroke is a multifaceted pathological condition driven by a complex interplay of multiple factors, including aberrant immune activation, chronic inflammatory cascades, dysregulated lipid metabolism, and synergistic effects of various risk factors. It can be categorized into several subtypes: large artery atherosclerosis (LAA), cardioembolism (CE), small artery occlusion (SAO), stroke of other determined etiology (SOE), and stroke of undetermined etiology (SUE) (2). Among these, LAA is the most prevalent subtype and is characterized as an immune-mediated chronic inflammatory process occurring within the arterial wall (3). Dysfunction in nutritional and metabolic pathways may disrupt immune homeostasis, promote chronic inflammation, and heighten susceptibility to immune-related disorders. According to anatomical location and vascular origin, cerebral vessels can be classified as intracranial, extracranial, anterior circulation and posterior circulation. Posterior circulation stroke, primarily resulting from lesions in the vertebrobasilar arterial system, is often insidious in onset and associated with poor clinical outcomes and high mortality rates (4, 5). In contrast to the more recognizable manifestations of anterior circulation stroke, such as hemiparesis or aphasia, early symptoms of posterior circulation stroke frequently present as dizziness, which may delay timely diagnosis and treatment. Therefore, the early identification and monitoring of vascular stenosis across intracranial and extracranial regions, as well as within both anterior and posterior circulations, are essential for improving outcomes.

Albumin-to-globulin ratio (AGR) is a metabolic biomarker that reflect nutritional and immune status. BMI is a globally recognized screening index for obesity and serves as an established risk factor for ischemic stroke (6, 7). Serum albumin is commonly used as a biochemical marker for evaluating nutritional status; reduced albumin levels may indicate malnutrition or compromised health. Globulin levels are closely associated with the degree of systemic inflammation. In addition to the metabolic burden caused by the inflammatory response itself, increased capillary permeability during inflammation can facilitate the extravasation of serum albumin into interstitial spaces, resulting in hypoproteinemia, which has been linked to adverse clinical outcomes (8). Compared with individual measurements of serum albumin or globulin, AGR demonstrates greater diagnostic and prognostic value (9). Emerging evidence suggests that AGR is associated with depression (10), frailty (11), prognosis in malignant tumors (12), chronic disease progression, and the risk of postoperative infections. A prospective cohort study demonstrated that, among patients with acute ischemic stroke, a lower serum albumin-to-globulin ratio was independently associated with an increased risk of poor functional outcome and all-cause mortality at both 3-month and 1-year follow-up (13). The study by Han et al. (14) demonstrated that a lower albumin-to-globulin ratio is associated with more pronounced carotid atherosclerosis; in acute ischemic stroke, the albumin-to-globulin ratio serves as a superior predictor of “vascular aging” compared with globulin alone. A large-scale population study based on NHANES further confirmed that individuals with lower AGR levels exhibit increased incidence of both hemorrhagic and ischemic stroke (15).

However, it remains unclear whether AGR has a direct association with the risk of cerebral atherosclerotic stenosis. To address this gap, the present study employed digital subtraction angiography (DSA) to investigate the potential relationship between serum AGR levels and cerebral atherosclerotic stenosis, including both intracranial and extracranial involvement, as well as lesions located in the anterior or posterior circulation. Stratified analyses were further conducted based on BMI, age, and sex, aiming to provide evidence that may inform future research on the risk stratification and etiology of cerebrovascular stenosis.

## Methods

### Study population

This retrospective observational study enrolled 1,189 adult patients diagnosed with acute ischemic stroke (AIS) who underwent DSA in the First Hospital of Jilin University between March 2024 and June 2025. The exclusion criteria were strictly defined as follows: (1) age <18 years; (2) presence of concurrent hemorrhagic stroke, cerebral aneurysm, or intracranial space-occupying lesions; (3) cardiogenic cerebral embolism, vascular malformations (including arteriovenous malformations or arteriovenous fistulas), or vasculitis; (4) severe hepatic or renal dysfunction, or active systemic infection; (5) coexisting hematological disorders, history of malignancy, or tuberculosis; (6) autoimmune diseases, immunodeficiency conditions, or ongoing immunosuppressive therapy (including glucocorticoids); (7) incomplete clinical records, including missing data on height, weight, plasma albumin, or globulin levels. The study protocol was reviewed and approved by the Medical Ethics Committee of the First Hospital of Jilin University (approval number: 2025-480).

### Clinical and laboratory data collection

Baseline data encompassed demographic characteristics, clinical parameters, and laboratory measurements. Specifically, the following variables were collected: age, sex, BMI (kg/m^2^), systolic blood pressure (SBP), diastolic blood pressure (DBP), history of comorbidities (including hypertension, diabetes mellitus, coronary heart disease (CHD), and stroke), smoking status, alcohol consumption history, and medication use (such as antihypertensive agents, hypoglycemic agents, statins, and antiplatelet drugs). Fasting venous blood samples were collected from all participants within 24 h of hospital admission. Laboratory assessments included white blood cell count (WBC), red blood cell count (RBC), hemoglobin (HGB), platelet count (PLT), alanine aminotransferase (ALT), aspartate aminotransferase (AST), albumin, globulin, fasting blood glucose, total cholesterol (TC), triglycerides (TG), high-density lipoprotein cholesterol (HDL-C), low-density lipoprotein cholesterol (LDL-C), homocysteine, creatinine, uric acid, and urea. The AGR was defined as the ratio of serum albumin (g/L) to globulin (g/L). Serum albumin was measured using the Bromocresol Green method, and serum globulin level was quantitatively determined by immunoturbidimetry. Rigorous quality control procedures were implemented, including daily internal quality control (with a coefficient of variation < 5% for all assays) and regular participation in external quality assessment programs.

### Imaging data acquisition and analysis

Cerebral angiography data were independently interpreted and subjected to quality control by two experienced neurointerventional specialists, each with over 5 years of diagnostic experience in DSA. Any disagreements in the quantitative stenosis measurement (defined as an interrater difference >10%) were adjudicated by a third senior neurointerventionalist who was blinded to the initial assessments. The vascular evaluation encompassed the following anatomical regions:

Intracranial arteries: M1–M2 segments of the middle cerebral artery, C6–C7 segments of the internal carotid artery, A1–A2 segments of the anterior cerebral artery, the entire basilar artery, V4 segment of the vertebral artery, and P1–P2 segments of the posterior cerebral artery; Extracranial arteries: the full length of the common carotid artery, C1–C5 segments of the internal carotid artery, V1–V3 segments of the vertebral artery, and the proximal portion of the subclavian artery; Anterior circulation: the common carotid artery – internal carotid artery – anterior/middle cerebral artery system; Posterior circulation: the vertebral artery – basilar artery – posterior cerebral artery system, including the origin of the subclavian artery.

The definition of a symptomatic stenosis was predicated on the occurrence of a transient ischemic attack or ischemic stroke within the relevant vascular territory in the month prior to DSA, with diagnoses adhering to ICD-11 standards (16). The degree of arterial stenosis was quantitatively assessed on DSA images using the North American Symptomatic Carotid Endarterectomy Trial (NASCET) criteria. Briefly, the stenosis percentage was calculated as: [1 – (minimal residual lumen diameter/normal distal internal carotid artery diameter)] × 100%. A stenosis was considered hemodynamically significant if the lumen reduction was ≥50%. Utilizing this metric, cases were categorized: a lumen reduction of ≥30% indicated stenosis, a reduction of 70–99% or occlusion indicated severe stenosis.

### Statistical analyses

All statistical analyses were conducted using R software (version 4.3.3), SPSS (version 26.0), and GraphPad Prism (version 9.0.0). Statistical significance was defined as a two-tailed *p*-value < 0.05.

The study population characteristics were divided into four groups based on AGR quartiles (Q1–Q4). The basic characteristics of the participants are presented as mean ± standard deviation or medians (25th–75th percentiles) for continuous variables and counts (percentages) for categorical variables. Comparisons among the four groups were conducted via analysis of variance (ANOVA) or the Kruskal–Wallis test for continuous variables and the chi-square (*χ*^2^) test for categorical variables. Multivariate logistic regression was used to analyze the associations between the AGR and the risk of various cerebrovascular stenosis, including anterior circulation stenosis, posterior circulation stenosis, intracranial stenosis and extracranial stenosis. In the quartile analyses, the *p*-value for trend was assessed by assigning the median value of the AGR to each quartile and treating this median as a continuous variable in the regression model. Model 1 was unadjusted; Model 2 was adjusted for age and sex; and Model 3 was further adjusted for age, sex, BMI, hypertension, diabetes mellitus, coronary heart disease, stroke, smoking and drinking. Multicollinearity among the covariates in the multivariate logistic regression models was evaluated using the variance inflation factor (VIF). A VIF value of less than 5 was considered to indicate no substantial multicollinearity. For each of the three models, *p*-values from all comparisons were adjusted for multiple testing using the Benjamini–Hochberg procedure to control the false discovery rate (FDR). Furthermore, restricted cubic spline (RCS) analyses with three knots was employed to examine potential non-linear relationships between AGR and different types of cerebrovascular stenosis risk. The location of three knots is based on clinical cut-offs. According to BMI status, the population was classified into two categories: individuals with a BMI ranging from 18.5 to 23.9 were defined as “normal weight,” those with a BMI of 24 or higher were defined as “overweight/obese.” We further stratified all participants by BMI, age (65 was the cutoff) and sex to explore the association between AGR and risk of cerebral stenosis in different states.

## Results

### Baseline characteristics of the study participants

**Figure 1:**
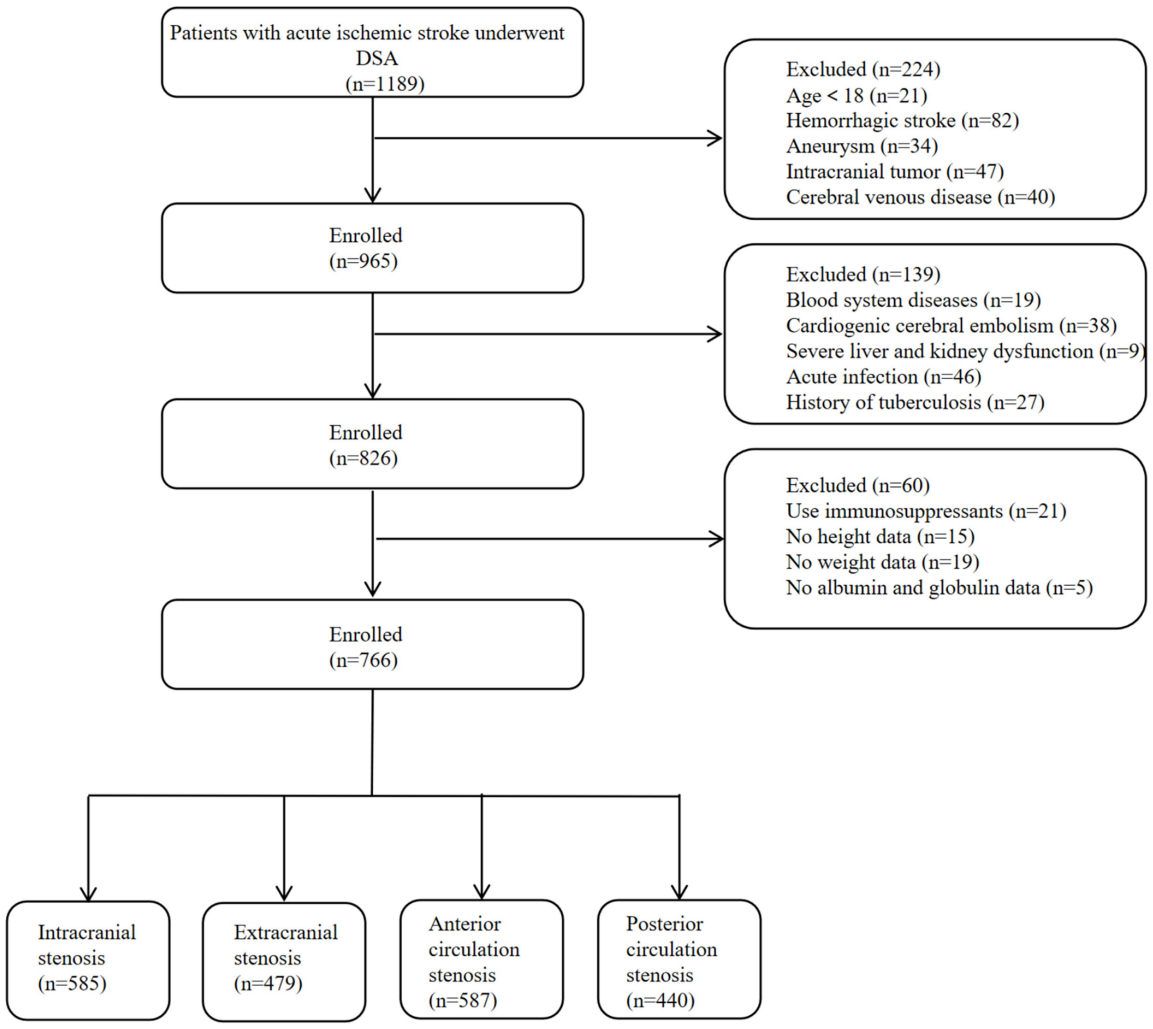
Flowchart of participants’ selection. DSA, digital subtraction angiography.

This study enrolled 1,189 patients with acute ischemic stroke (AIS) who underwent digital subtraction angiography (DSA), and 766 cases completed the data analysis finally (enrollment process is shown in Figure 1).

Patients were divided into four groups according to AGR levels: Q1 group (AGR ≤ 1.34), Q2 group (1.34 < AGR ≤ 1.49), Q3 group (1.49 < AGR ≤ 1.64), and Q4 group (AGR > 1.64). Baseline characteristics are shown in Table 1: the mean age of patients was 63.6 years, and 50.1% were male. Hypertension was present in 514 cases (67.1%). Among all patients, 587 cases (76.6%) had anterior circulation stenosis, 440 cases (57.4%) had posterior circulation stenosis, 585 cases (76.4%) had intracranial vascular stenosis, and 479 cases (62.5%) had extracranial cerebrovascular stenosis. Additionally, the incidences of posterior circulation stenosis and intracranial stenosis were lower in the Q4 group.

**Table 1:** Baseline characteristics of participants categorized by AGR quartiles.

Variables	Overall	Q1	Q2	Q3	Q4	*p*-value
	(*n* = 766)	(*n* = 197)	(*n* = 189)	(*n* = 189)	(*n* = 191)	
Age (years)	63.6 ± 11.6	65.3 ± 11.2	65.7 ± 14.1	61.9 ± 10.2	61.5 ± 9.6	<0.001***
sex (Male)	384 (50.1)	84 (42.6)	81 (42.9)	100 (52.9)	119 (62.3)	<0.001***
BMI (kg/m^2^)	24.9 ± 3.9	24.9 ± 4.3	25.2 ± 4.2	25.0 ± 3.8	24.7 ± 3.4	0.609
SBP	146.9 ± 20.2	149.3 ± 22.5	146.9 ± 19.7	146.7 ± 19.1	144.6 ± 19.2	0.149
DBP	85.7 ± 12.6	87.6 ± 14.0	84.8 ± 12.3	85.2 ± 10.6	85.5 ± 13.0	0.115
Medical history						
Hypertension	514 (67.1)	136 (69.0)	129 (68.3)	122 (64.6)	127 (66.5)	0.792
DM	223 (29.1)	58 (29.4)	51 (27.0)	51 (27.0)	63 (33.0)	0.527
CHD	157 (20.5)	44 (22.3)	38 (20.1)	42 (22.2)	33 (17.3)	0.575
Stroke	239 (31.2)	66 (33.5)	62 (32.8)	59 (31.2)	52 (27.2)	0.548
Smoking	170 (22.2)	44 (22.3)	37 (19.6)	44 (23.3)	45 (23.6)	0.780
Drinking	60 (7.8)	21 (10.7)	10 (5.3)	13 (6.9)	16 (8.4)	0.242
Medication						
Antihypertensive drugs	444 (58.0)	119 (60.4)	115 (60.8)	104 (55.0)	106 (55.5)	0.518
Antidiabetic drugs	204 (26.6)	54 (27.4)	45 (23.8)	48 (25.4)	57 (29.8)	0.575
Statins	171 (22.3)	47 (23.9)	48 (25.4)	41 (21.7)	35 (18.3)	0.376
Antiplatelet aggregation drugs	292 (38.1)	76 (38.6)	78 (41.3)	75 (39.7)	63 (33.0)	0.370
Laboratory tests						
WBC (×10^9^/L)	6.2 (5.2, 7.8)	6.4 (5.2, 8.0)	6.0 (5.3, 8.0)	6.2 (5.2, 7.8)	6.1 (5.3, 7.6)	0.796
RBC (×10^12^/L)	4.4 (3.7, 4.9)	4.3 (3.8, 5.0)	4.3 (3.6, 4.9)	4.4 (3.6, 5.0)	4.4 (3.8, 4.9)	0.397
HGB (g/L)	140.0 ± 14.6	140.2 ± 13.5	138.7 ± 15.4	139.4 ± 14.9	141.5 ± 14.7	0.282
PLT (×10^9^/L)	224.1 ± 51.9	224.1 ± 50.1	224.6 ± 50.2	223.4 ± 53.5	224.0 ± 54.1	0.997
ALT (U/L)	19.6 (16.8, 24.8)	20.9 (16.3, 25.2)	19.6 (17.0, 25.6)	19.6 (17.0, 24.3)	19.1 (16.3, 24.0)	0.604
AST (U/L)	17.5 (14.0, 22.4)	17.5 (13.0, 22.4)	17.5 (14.3, 19.8)	17.1 (13.7, 21.6)	18.2 (15.1, 23.9)	0.204
Albumin (g/L)	40.1 (37.6, 43.1)	38.8 (35.4, 41.2)	39.5 (37.3, 43.0)	40.7 (38.7, 43.9)	41.5 (39.5, 44.7)	<0.001***
Glucose (mmol/L)	5.9 (5.2, 7.1)	5.9 (5.3, 7.2)	5.8 (5.2, 6.9)	5.9 (5.2, 7.1)	5.8 (5.1, 7.5)	0.354
TG (mmol/L)	1.4 (1.0, 1.8)	1.4 (1.1, 2.0)	1.4 (1.1, 1.7)	1.3 (1.0, 1.7)	1.3 (0.9, 1.8)	0.081
TC (mmol/L)	4.0 (3.4, 4.9)	4.3 (3.6, 5.2)	4.1 (3.4, 5.0)	3.9 (3.3, 4.8)	4.0 (3.3, 4.6)	<0.001***
HDL-C (mmol/L)	1.1 (0.9, 1.3)	1.1 (0.9, 1.2)	1.1 (0.9, 1.3)	1.1 (0.9, 1.3)	1.0 (0.9, 1.2)	0.923
LDL-C (mmol/L)	2.5 (2.0, 3.1)	2.8 (2.2, 3.3)	2.6 (2.0, 3.1)	2.3 (1.8, 3.0)	2.4 (1.9, 2.9)	<0.001***
Homocysteine (μmmol/L)	11.5 (9.2, 14.7)	12.2 (9.3, 15.6)	11.6 (9.3, 14.4)	11.1 (8.7, 13.9)	11.3 (9.5, 14.7)	0.084
Uric acid (μmol/L)	307.1 ± 81.5	306.9 ± 89.6	306.6 ± 75.4	308.1 ± 84.6	306.7 ± 76.0	0.998
Urea (mmol/L)	6.2 (5.2, 7.7)	6.0 (5.2, 7.6)	6.1 (5.2, 7.6)	6.4 (5.2, 7.7)	6.4 (5.4, 7.8)	0.567
Creatinine (μmol/L)	66.5 (56.2, 78.5)	67.1 (57.6, 77.5)	66.1 (56.1, 81.6)	65.8 (54.7, 79.4)	66.6 (56.1, 78.4)	0.943
Stenosis status						
No stenosis	46 (6.0)	8 (4.1)	9 (4.8)	13 (6.9)	16 (8.4)	0.262
Intracranial stenosis	585 (76.4)	159 (80.7)	151 (79.9)	142 (75.1)	133 (69.6)	0.040*
Extracranial stenosis	479 (62.5)	129 (65.5)	116 (61.4)	115 (60.8)	119 (62.3)	0.784
Anterior circulation stenosis	587 (76.6)	153 (77.7)	143 (75.7)	143 (75.7)	148 (77.5)	0.941
Posterior circulation stenosis	440 (57.4)	130 (66.0)	109 (57.7)	99 (52.4)	102 (53.4)	0.027*

AGR, Albumin/Globulin Ratio; BMI, body mass index; SBP, systolic blood pressure; DBP, diastolic blood pressure; DM, diabetes mellitus; CHD, coronary heart disease; WBC, white blood cell count; RBC, red blood cell count; HGB, Hemoglobin; PLT, platelet count; ALT, alanine aminotransferase; AST, aspartate transaminase; TG, triglyceride; TC, total cholesterol; HDL-C, high-density lipoprotein cholesterol; LDL-C, low-density lipoprotein cholesterol. Smoking status: Patients were categorized as ‘Yes’ (smokers) if they were current smokers (defined as having smoked cigarettes continuously or cumulatively for ≥6 months and were smoking at the time of or within 1 month prior to admission). All other patients were classified as ‘No’ (non-smokers); Alcohol use: Patients were categorized as ‘Yes’ (drinkers) if they were current alcohol drinkers (defined as consuming alcohol at least once per week for a continuous period of ≥6 months prior to admission). All other patients were classified as ‘No’ (non-drinkers); Stroke history: A history of stroke was confirmed by a combination of self-report and verification through medical records, requiring documented clinical evidence (e.g., neurological deficit) supported by compatible neuroimaging findings (CT or MRI). *p < 0.05, ***p < 0.001.

**Figure 2:**
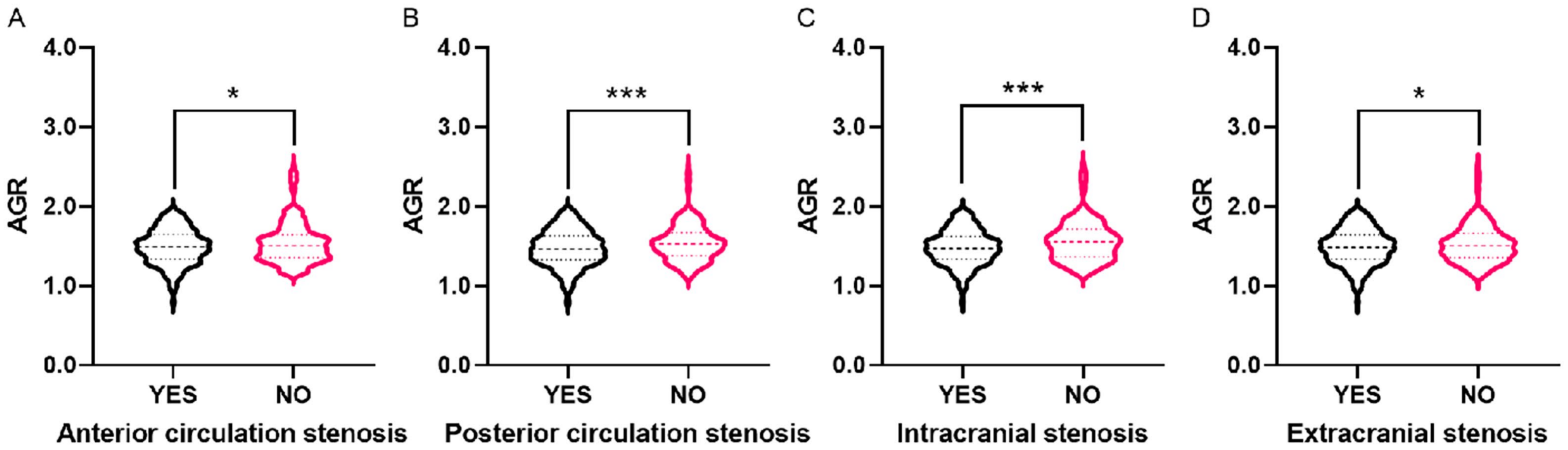
The violin plots demonstrating the distribution of the AGR among participants in different groups. **(A)** With and without anterior circulation stenosis. **(B)** With and without posterior circulation stenosis. **(C)** With and without intracranial stenosis. **(D)** With and without extracranial stenosis. The AGR is a dimensionless quantitative variable. The demarcation values used throughout the analysis are as follows: Q1 (≤1.34), Q2 (1.34 < AGR ≤ 1.49), Q3 (1.49 < AGR ≤ 1.64), Q4 (>1.64). AGR, Albumin/Globulin Ratio. *p < 0.05, ***p < 0.001.

Violin plots showed differences in AGR distribution among patients with different stenosis statuses. AGR levels in patients with anterior circulation stenosis, posterior circulation stenosis, intracranial vascular stenosis, and extracranial cerebrovascular stenosis were all lower than those in participants without corresponding vascular stenosis (Figure 2).

### The association of AGR with anterior and posterior circulation stenosis

As shown in Table 2, after adjusting for covariates (age, sex, BMI, hypertension, diabetes mellitus, CHD, stroke history, smoking, and drinking), no significant association was observed between AGR and anterior circulation stenosis when AGR was analyzed as either a continuous or categorical variable (all *p* > 0.05). However, after adjusting for the same covariates, a negative association was observed between AGR and the occurrence of posterior circulation stenosis when AGR was analyzed as a continuous variable (OR = 0.27, 95% CI = 0.14 ~ 0.53, *p* < 0.001). When AGR was analyzed as a categorical variable, compared with patients in the lowest AGR group (Q1), those in the higher AGR groups (Q3: OR = 0.60, 95% CI = 0.39 ~ 0.92, *p* = 0.019; Q4: OR = 0.59, 95% CI = 0.38 ~ 0.90, *p* = 0.015) had a significantly lower incidence of posterior circulation stenosis. Furthermore, it is noteworthy that AGR quartiles were significantly associated with a trend in posterior circulation stenosis (*p* for trend < 0.05). We assessed multicollinearity among all covariates included in the multivariate logistic regression models (Model 3) by calculating the VIF. The results confirm that no significant multicollinearity was present (all VIF values < 5) (Supplementary Table S1). Then, we applied the Benjamini-Hochberg correction for multiple testing to adjust the *p*-values for all comparisons across the three models (Supplementary Table S2).

**Table 2:** Association of AGR with anterior and posterior circulation stenosis.

Variables	Model 1	*p*-value	Model 2	*p*-value	Model 3	*p*-value
	OR (95% CI)		OR (95% CI)		OR (95% CI)	
Anterior circulation stenosis						
AGR	0.46 (0.22 ~ 0.94)	0.034*	0.51 (0.25 ~ 1.07)	0.076	0.49 (0.23 ~ 1.23)	0.060
AGR quartiles						
Q1	Reference					
Q2	0.89 (0.56 ~ 1.43)	0.642	0.89 (0.55 ~ 1.43)	0.630	0.89 (0.55 ~ 1.43)	0.628
Q3	0.89 (0.56 ~ 1.43)	0.642	0.95 (0.59 ~ 1.53)	0.835	0.94 (0.58 ~ 1.52)	0.799
Q4	0.99 (0.61 ~ 1.60)	0.966	1.08 (0.66 ~ 1.76)	0.758	1.06 (0.65 ~ 1.72)	0.832
*P* for trend		0.998		0.682		0.738
Posterior circulation stenosis						
AGR	0.28 (0.15 ~ 0.53)	<0.001***	0.28 (0.14 ~ 0.55)	<0.001***	0.27 (0.14 ~ 0.53)	<0.001***
AGR quartiles						
Q1	Reference					
Q2	0.70 (0.47 ~ 1.06)	0.093	0.70 (0.46 ~ 1.06)	0.090	0.71 (0.47 ~ 1.09)	0.115
Q3	0.57 (0.38 ~ 0.86)	0.007**	0.59 (0.39 ~ 0.89)	0.013*	0.60 (0.39 ~ 0.92)	0.019*
Q4	0.59 (0.39 ~ 0.89)	0.012*	0.60 (0.39 ~ 0.91)	0.016*	0.59 (0.38 ~ 0.90)	0.015*
*P* for trend		0.010*		0.016*		0.016*

Model 1: unadjusted; Model 2:adjusted for age and sex; Model 3:adjusted for age, sex, BMI, hypertension, diabetes mellitus, coronary heart disease, stroke, smoking and drinking. AGR, Albumin/Globulin Ratio; BMI, body mass index; OR, odds ratio; CI, confidence interval. *p < 0.05, **p < 0.01, ***p < 0.001.

**Figure 3:**
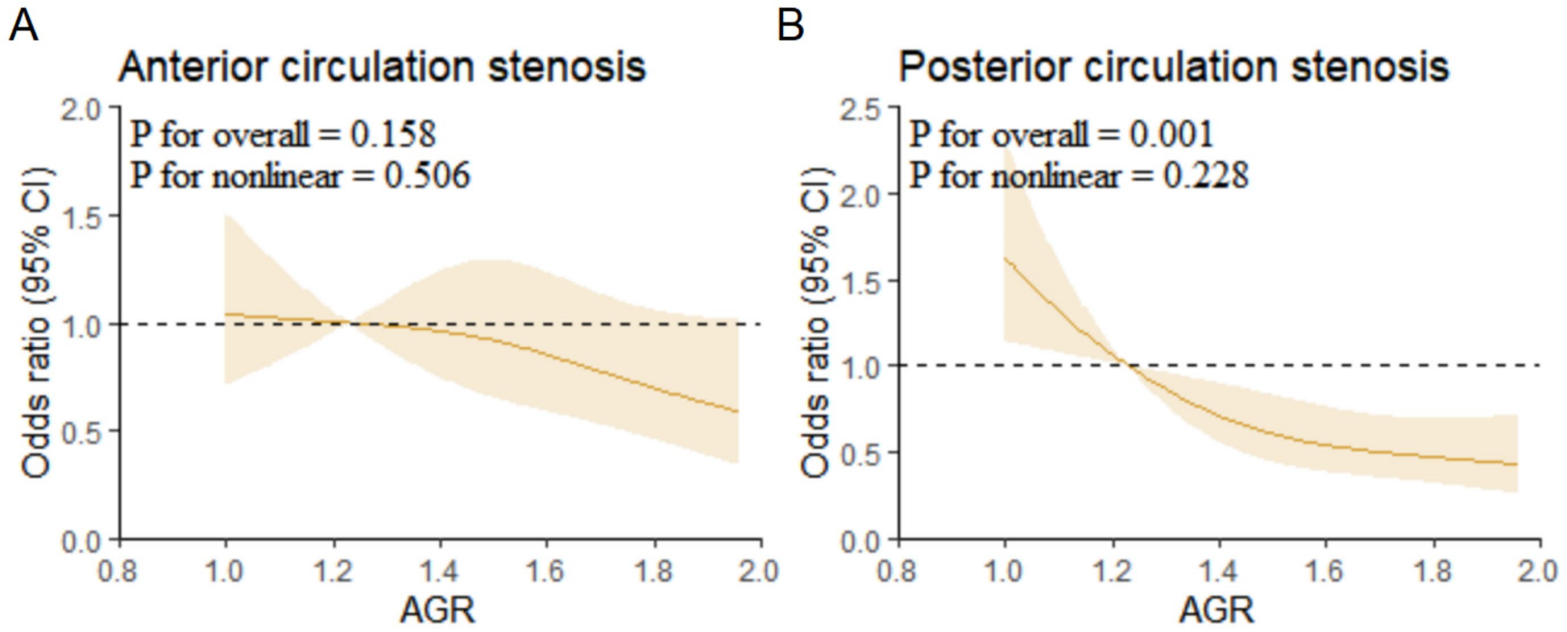
Restricted cubic spline regression analysis for association of AGR with anterior and posterior circulation stenosis. **(A)** Anterior circulation stenosis. **(B)** Posterior circulation stenosis. AGR, Albumin/Globulin Ratio.

Multivariable-adjusted restricted cubic spline (RCS) analysis showed a linear association between AGR and posterior circulation stenosis (*p* for overall = 0.001, *p* for non-linear = 0.228) (Figure 3).

### The association of AGR with intracranial and extracranial stenosis

After adjusting for covariates, when AGR was analyzed as a continuous variable, a negative association was observed between AGR and intracranial stenosis (OR = 0.20, 95% CI = 0.09 ~ 0.42, *p* < 0.001), as well as extracranial stenosis (OR = 0.47, 95% CI = 0.24 ~ 0.93, *p* = 0.029). When AGR was analyzed as a categorical variable, compared with patients in the lowest AGR group (Q1), those in the higher AGR groups (Q4: OR = 0.55, 95% CI = 0.34 ~ 0.89, *p* = 0.015) had a significantly lower incidence of intracranial circulation stenosis. Moreover, it is noteworthy that AGR quartiles were significantly associated with a trend in intracranial stenosis (*p* for trend < 0.05). However, when AGR was analyzed as a categorical variable, no association was observed between AGR and extracranial stenosis (Table 3).

**Table 3:** Association of AGR with intracranial and extracranial stenosis.

Variables	Model 1	*p*-value	Model 2	*p*-value	Model 3	*p*-value
	OR (95% CI)		OR (95% CI)		OR (95% CI)	
Intracranial stenosis						
AGR	0.21 (0.10 ~ 0.43)	<0.001***	0.20 (0.09 ~ 0.43)	<0.001***	0.20 (0.09 ~ 0.42)	<0.001***
AGR quartiles						
Q1	Reference					
Q2	0.95 (0.58 ~ 1.57)	0.840	0.96 (0.58 ~ 1.58)	0.859	0.93 (0.56 ~ 1.54)	0.774
Q3	0.72 (0.45 ~ 1.17)	0.187	0.72 (0.44 ~ 1.17)	0.178	0.70 (0.43 ~ 1.15)	0.162
Q4	0.55 (0.34 ~ 0.88)	0.012*	0.55 (0.34 ~ 0.86)	0.014*	0.55 (0.34 ~ 0.89)	0.015*
*P* for trend		0.005**		0.007**		0.006**
Extracranial stenosis						
AGR	0.48 (0.25 ~ 0.91)	0.024*	0.48 (0.25 ~ 0.94)	0.033*	0.47 (0.24 ~ 0.93)	0.029*
AGR quartiles						
Q1	Reference					
Q2	0.84 (0.55 ~ 1.27)	0.402	0.84 (0.55 ~ 1.28)	0.411	0.87 (0.56 ~ 1.33)	0.508
Q3	0.82 (0.54 ~ 1.24)	0.345	0.87 (0.57 ~ 1.33)	0.520	0.88 (0.57 ~ 1.36)	0.565
Q4	0.87 (0.58 ~ 1.32)	0.515	0.89 (0.58 ~ 1.37)	0.586	0.88 (0.57 ~ 1.35)	0.550
*P* for trend		0.551		0.663		0.715

Model 1: unadjusted; Model 2:adjusted for age and sex; Model 3:adjusted for age, sex, BMI, hypertension, diabetes mellitus, coronary heart disease, stroke, smoking and drinking. AGR, Albumin/Globulin Ratio; BMI, body mass index; OR, odds ratio; CI, confidence interval. *p < 0.05, **p < 0.01, ***p < 0.001.

**Figure 4:**
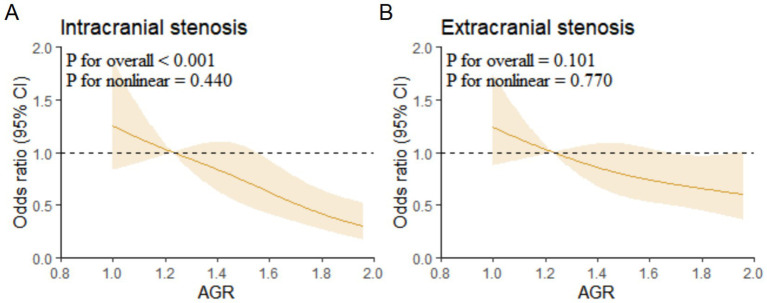
Restricted cubic spline regression analysis for association of AGR with intracranial and extracranial stenosis. **(A)** Intracranial stenosis. **(B)** Extracranial stenosis. AGR, Albumin/Globulin Ratio.

Multivariable-adjusted RCS analysis showed a linear association between AGR and intracranial vascular stenosis (*p* for overall < 0.001, *p* for non-linear = 0.440) (Figure 4).

### The association between AGR and vascular stenosis stratified by BMI

**Figure 5:**
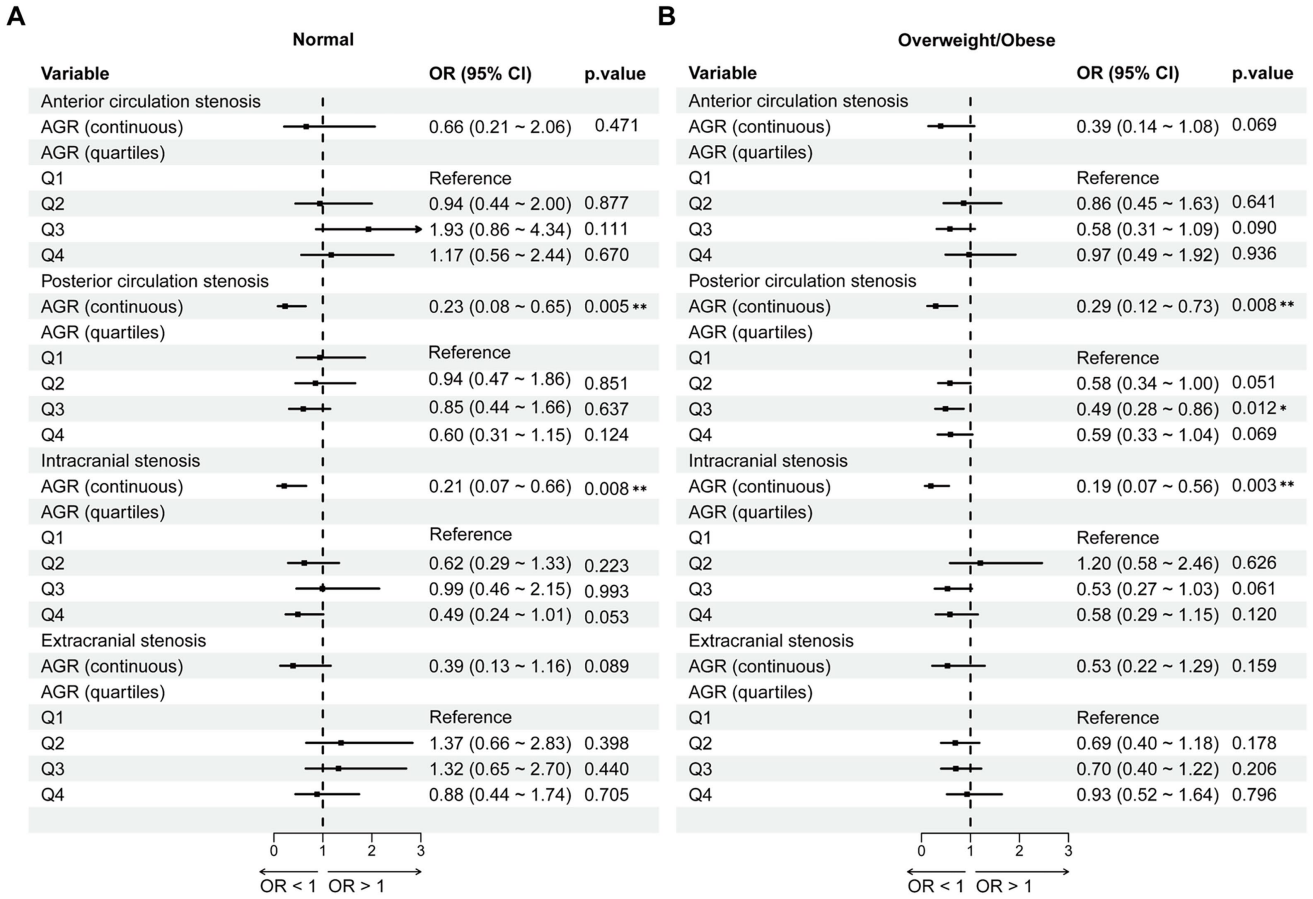
The association between AGR and vascular stenosis stratified by BMI. **(A)** Normal weight group. **(B)** Overweight/Obese group. Adjusted for age, sex, hypertension, diabetes mellitus; coronary heart disease, stroke, smoking and drinking. AGR, Albumin/Globulin Ratio; OR, odds ratio; CI, confidence interval. *p < 0.05, **p < 0.01.

We further performed stratified analysis by BMI status to explore the association between AGR and cerebrovascular stenosis (Figure 5). Firstly, when AGR was treated as a continuous variable, AGR was negatively associated with the occurrence of posterior circulation stenosis (normal weight: OR = 0.23, 95%CI = 0.08 ~ 0.65, *p* = 0.005; overweight/obese: OR = 0.29, 95%CI = 0.12 ~ 0.73, *p* = 0.008) and intracranial vascular stenosis (normal weight: OR = 0.21, 95%CI = 0.07 ~ 0.66, *p* = 0.008; overweight/obese: OR = 0.19, 95%CI = 0.07 ~ 0.56, *p* = 0.003) in both normal weight and overweight/obese groups.

When AGR was categorized, compared with the Q1 group, only AGR in the Q3 group of the overweight/obese group was negatively associated with posterior circulation stenosis (OR = 0.49, 95%CI = 0.28 ~ 0.86, *p* = 0.012). Overall, the association between AGR and cerebrovascular stenosis was hardly affected by BMI status.

### The association between AGR and vascular stenosis stratified by age and sex

Subgroup analyses were further performed to evaluate the predictive value of AGR for cerebrovascular stenosis in different populations. Specifically, when stratified by age (cutoff at 65 years), as a continuous variable, AGR was negatively associated with posterior circulation stenosis (OR = 0.39, 95% CI = 0.16 ~ 0.96, *p* = 0.041) and intracranial stenosis (OR = 0.23, 95% CI = 0.08 ~ 0.66, *p* = 0.006) in participants <65 years, and similarly with posterior circulation stenosis (OR = 0.17, 95% CI = 0.06 ~ 0.48, *p* = 0.001) and intracranial stenosis (OR = 0.14, 95% CI = 0.05 ~ 0.41, *p* < 0.001) in those ≥65 years. As a categorical variable, in participants ≥65 years, compared with the Q1 group, the Q4 group showed a negative association between AGR and posterior circulation stenosis (OR = 0.52, 95% CI = 0.27 ~ 0.99, *p* = 0.047), as well as intracranial stenosis (OR = 0.32, 95% CI = 0.16 ~ 0.65, *p* = 0.002) (Supplementary Table S3).

When stratified by sex, as a continuous variable, AGR was negatively associated with posterior circulation stenosis (OR = 0.35, 95% CI = 0.14 ~ 0.88, *p* = 0.025) and intracranial stenosis (OR = 0.16, 95% CI = 0.06 ~ 0.45, *p* = 0.001) in males, and with posterior circulation stenosis (OR = 0.22, 95% CI = 0.08 ~ 0.60, *p* = 0.003) and intracranial stenosis (OR = 0.22, 95% CI = 0.07 ~ 0.71, *p* = 0.011) in females (Supplementary Table S4). Overall, the results of age- and sex-stratified analyses were consistent with those in the overall population.

## Discussion

In this study, we observed that the AGR was negatively associated with the presence of cerebral atherosclerotic stenosis, including both anterior and posterior circulation stenosis as well as intracranial and extracranial stenosis. This association remained consistent across subgroups stratified by BMI, age, and sex. Furthermore, our findings revealed, for the first time, a linear relationship between AGR levels and the severity of posterior circulation and intracranial stenosis. These results suggest that AGR may serve as a novel biomarker for cerebral artery stenosis and could potentially offer enhanced predictive value for stenosis risk in specific vascular territories.

Albumin is a plasma protein synthesized by the hepatic tissue and serves as a critical component in maintaining colloid osmotic pressure and facilitating the transport of various substances within the circulatory system. It exerts multiple physiological functions, including modulation of immune responses, mitigation of inflammatory injury, and scavenging of free radicals (17). Globulin, predominantly synthesized by lymphoid organs, is primarily secreted by plasma cells. As a central element of humoral immunity, globulin collaborates with albumin to preserve plasma oncotic pressure, thereby preventing tissue edema. Additionally, it plays an essential role in complement activation and inflammatory processes. Under conditions of trauma, inflammation, or systemic stress, serum albumin levels are typically diminished. A reduction in albumin concentration (<35 g/L) reflects impaired hepatic synthetic capacity and compromised nutritional status. Consequently, several studies have proposed that serum albumin may serve as a potential biomarker for assessing systemic inflammatory states (15, 18).

Clinical observational studies have demonstrated that persistent hypoalbuminemia is associated with adverse outcomes in patients with sepsis, whereas normalization of serum albumin levels may indicate clinical improvement (19). Accumulating evidence indicates that serum albumin levels significantly influence clinical outcomes in individuals with ischemic stroke (20). A nationwide registry study involving 13,618 patients revealed that elevated admission albumin levels were independently linked to improved functional recovery and reduced mortality, with consistent associations observed at both 3-month and 1-year follow-up periods (21). Similarly, a meta-analysis encompassing 23,597 patients confirmed that low admission albumin levels were significantly correlated with increased long-term mortality following stroke (22). Nonetheless, the therapeutic use of albumin infusion during the acute phase of ischemic stroke remains controversial. Some studies suggest that such treatment may elevate the risk of pulmonary edema and intracranial hemorrhage (23), although these effects appear to be influenced by the dosage and timing of administration.

AGR represents a novel biomarker reflecting both nutritional status and immune function, which has demonstrated significant clinical relevance in the pathogenesis and progression of various diseases. Compared to individual measurements of albumin or globulin levels, AGR exhibits superior prognostic performance, likely due to its integrated assessment of both nutritional and inflammatory components that influence disease outcomes. Currently, AGR is recognized as a valuable prognostic indicator in multiple malignancies. A meta-analysis summarizing 18 studies on solid tumors over the past decade reported that AGR was significantly associated with at least one prognostic outcome in each cancer type examined, supporting its role as a cost-effective and clinically meaningful predictive marker (12). When AGR is less than 1.1, it is associated with a shorter overall survival period in patients with renal cell carcinoma (24). Moreover, emerging evidence suggests that AGR serves as an effective diagnostic biomarker for periprosthetic joint infection following prosthetic implantation (25). In critically ill ICU patients, reduced AGR levels are independently associated with increased risks of pneumonia and 28-day mortality (26). A cross-sectional study involving 8,381 participants revealed that long-term regular aerobic exercise (≥2 sessions per week for more than 1 year) significantly elevates AGR levels, potentially contributing to the inhibition of atherosclerotic progression. Furthermore, higher AGR values were correlated with reduced systemic inflammation (27). Han et al. (14) reported a significant association between AGR and common carotid artery intima-media thickness among ischemic stroke patients, suggesting a potential role of AGR in modulating atherosclerosis progression. Specifically, elevated AGR levels were associated with a 59% reduction in the risk of abnormal intima-media thickness. However, the mere presence of atherosclerosis does not necessarily lead to hemodynamically significant stenosis, as this progression depends on plaque characteristics, spatial distribution, and vascular compensatory mechanisms. In the present study, we provide the first evidence demonstrating a direct negative correlation between AGR levels and the presence of cerebral atherosclerotic stenosis, encompassing both anterior and posterior circulation as well as intracranial and extracranial involvement. These findings suggest that individuals with low AGR may benefit from earlier vascular evaluation and intensified therapeutic interventions to prevent irreversible stenotic changes.

It is well known that being overweight and obese are risk factors for atherosclerosis. The immune response in atherosclerosis involves various immune cells, along with their associated cytokines and adhesion molecules. Cholesterol in the brain accounts for approximately 25% of the body’s total cholesterol, with the majority located in the myelin sheath (28). Cholesterol is also a key component of synaptic vesicles, playing essential roles in maintaining cell membrane integrity, myelin formation, synaptic development, neural repair and remodeling, and signaling pathways critical to neurodevelopment (29). However, when cholesterol becomes overloaded, it can aggregate into crystals that activate the NLRP3 inflammasome, a crucial driver of atherosclerosis (30, 31). Elevated circulating cholesterol levels could influence T-cell activation and reprogramming, promoting the differentiation of CD4 + T cells toward an inflammatory Th1 phenotype, as observed in mouse models (32). For individuals with a BMI indicating overweight status, early implementation of weight management strategies and active weight reduction interventions have been demonstrated to significantly lower the risk of ischemic stroke (6). Notably, an elevated BMI during adolescence in males has been linked to an increased risk of early-onset atrial fibrillation, which in turn is associated with worse long-term outcomes following ischemic stroke (7).

Emerging evidence also suggests that BMI may influence stroke incidence through interactive effects with the triglyceride-glucose index (TyG), highlighting the complex interplay between metabolic dysregulation and cerebrovascular events (33). Importantly, our findings reveal that the association between AGR and atherosclerotic vascular stenosis remains robust and consistently independent of confounding variables such as body mass index, age, or gender. This observation underscores the potential role of AGR as a stable and reliable biomarker across diverse patient subgroups. These results further imply that, while traditional risk factor modification remains crucial, clinicians should also monitor albumin and globulin levels in young individuals with normal body weight. Maintaining adequate intake of high-quality proteins to prevent hypoproteinemia may serve as a complementary strategy for reducing the risk of adverse clinical outcomes.

Furthermore, our study revealed a linear association between AGR levels and the presence of posterior circulation stenosis as well as intracranial vascular stenosis. This intriguing discrepancy may be attributed to a confluence of anatomical, hemodynamic, and pathological factors. Firstly, from an anatomical perspective, this phenomenon may be attributed to anatomical and physiological differences between intracranial and extracranial arteries, particularly in terms of nutrient vessel density and antioxidant capacity, which limit lipid deposition and render intracranial arteries more vulnerable to inflammatory insults, and intracranial arteries reside within the confined space of the skull and lack an external elastic lamina and vasa vasorum, rendering them more vulnerable to systemic metabolic and inflammatory insults reflected by AGR, compared to the more resilient extracranial arteries (34). Secondly, hemodynamic characteristics differ significantly; the posterior circulation, with its more tortuous anatomy and unique flow patterns (e.g., in the vertebrobasilar system), might experience altered shear stress that modulates the localization and progression of inflammation-driven atherosclerosis, to which AGR is intrinsically linked (13, 27). Lastly, posterior and anterior circulation arteries originate from distinct embryonic vascular systems, leading to inherent structural disparities in their vessel walls and more tortuous anatomical pathways in the posterior circulation (35). These morphological features directly influence cerebral hemodynamics (36) and may render posterior circulation vessels more sensitive to systemic nutritional status and localized arterial wall inflammation. High-Resolution Vessel Wall Imaging has further demonstrated marked differences in plaque morphology between anterior and posterior circulation arteries. Specifically, posterior circulation plaques exhibit larger surface areas and stronger contrast enhancement, likely due to increased inflammatory activity and neovascularization, thereby elevating the risk of plaque rupture (37). Compared with anterior circulation stroke, posterior circulation involvement is typically associated with more severe clinical presentations, worse functional outcomes, and higher mortality rates. The novel association between AGR and posterior circulation stenosis identified in this study may serve as a cost-effective and accessible parameter associated with the presence of posterior circulation stenosis. When integrated with conventional risk factors, AGR may aid in identifying asymptomatic high-risk individuals, thereby contributing to our understanding of the disease etiology and informing hypotheses for future interventional studies.

With the rapid advancement of multi-omics technologies, high-throughput analytical tools, and artificial intelligence, research into stroke biomarkers is transitioning from single-molecule approaches toward multidimensional and dynamic integration models. This paradigm shift presents both new opportunities and challenges for the precision diagnosis and tailored management of stroke. However, several limitations must be acknowledged. First, as a single-center investigation, our findings may be subject to selection bias and may not fully represent broader patient populations. Second, the current observational design does not allow for causal inference regarding the relationship between AGR and cerebral vascular stenosis. Further mechanistic studies and large-scale, multicenter prospective trials are warranted to validate these findings and assess their clinical utility. While our findings suggest the potential value of AGR in risk assessment, it is crucial to interpret these results within the context of our study population—individuals who have already experienced an acute ischemic stroke. Therefore, our results primarily illuminate the role of AGR in the pathophysiology of established cerebral atherosclerosis rather than its predictive power for first-ever events in asymptomatic individuals. The promising clinical outlook of using AGR for identifying asymptomatic high-risk individuals remains a hypothesis that must be rigorously validated in future large-scale, prospective cohort studies conducted in community-based populations.

## Conclusion

This study demonstrates a significant association between AGR levels and the presence of posterior circulation stenosis as well as intracranial vascular stenosis. As a readily accessible and cost-effective biomarker, AGR holds potential for the early identification of cerebral atherosclerotic stenosis. Incorporating AGR into clinical practice may facilitate timely risk factor management and contribute to more informed decision-making in the diagnosis and treatment of stroke.

## Data Availability

The raw data supporting the conclusions of this article will be made available by the authors, without undue reservation.

## Glossary

AGR: Albumin-to-Globulin Ratio
AIS: Acute Ischemic Stroke
AST: Aspartate Transaminase
ALT: Alanine Aminotransferase
ANOVA: Analysis of Variance
BMI: Body Mass Index
CHD: Coronary Heart Disease
CE: Cardioembolism
CI: Confidence Interval
DM: Diabetes Mellitus
DBP: Diastolic Blood Pressure
HDL-C: High-Density Lipoprotein Cholesterol
HGB: Hemoglobin
LAA: Large Artery Atherosclerosis
LDL-C: Low-Density Lipoprotein Cholesterol
OR: Odds Ratio
PLT: Platelet Count
RCS: Restricted Cubic Spline
RBC: Red Blood Cell Count
SAO: Small Artery Occlusion
SOE: Stroke of Other Determined Etiology
SUE: Stroke of Undetermined Etiology
SBP: Systolic Blood Pressure
TG: Triglyceride
TC: Total Cholesterol
TyG: Triglyceride-Glucose Index
WBC: White Blood Cell Count
