# Supplementary material for: Association between serum albumin-to-globulin ratio and subtypes of cerebral atherosclerotic stenosis in acute ischemic stroke
Source: Front Neurol. 2025 Oct 16;16:1666940. doi: 10.3389/fneur.2025.1666940 (PMC12571648; doi:10.3389/fneur.2025.1666940)
Supplement: Supplementary file 1 [file Table_1.DOCX]

**Table S1** VIF analysis between AGR and other covariates

|  | VIF |
| --- | --- |
| Age | 1.147 |
| Sex | 1.079 |
| BMI | 1.082 |
| Hypertension | 1.047 |
| Diabetes mellitus | 1.033 |
| Coronary heart disease | 1.090 |
| Stroke | 1.061 |
| Smoking | 1.270 |
| Drinking | 1.162 |

VIF, variance inflation factor; BMI,body mass index; AGR, Albumin/Globulin Ratio.

**Table S2** The results of multiple comparisons using the Benjamini–Hochberg false discovery rate (FDR) procedure

|  | Model 1 |  | FDR-corrected  p-value |  | Model 2 |  | FDR-corrected  p-value |  | | Model 3 | |  | | FDR-corrected  p-value | | |  |
| --- | --- | --- | --- | --- | --- | --- | --- | --- | --- | --- | --- | --- | --- | --- | --- | --- | --- |
|  | OR (95% CI) |  |  |  | OR (95% CI) |  |  |  | | OR (95% CI) | |  | |  |  |  |  |
| **Anterior circulation stenosis** | | | | | | | | | | | | | | | | |  |
| AGR | 0.46 (0.22~0.94) |  | 0.068 |  | 0.51 (0.25~1.07) |  | 0.150 |  | | 0.49 (0.23~1.23) | |  | | 0.120 | | |  |
| AGR quartiles | | | | | | | | | | | | | | | | |  |
| Q1 | Reference |  |  |  |  |  |  |  | |  | |  | |  | | |  |
| Q2 | 0.89 (0.56~1.43) |  | 0.803 |  | 0.89 (0.55~1.43) |  | 0.835 |  | | 0.89 (0.55~1.43) | |  | | 0.832 | | |  |
| Q3 | 0.89 (0.56~1.43) |  | 0.803 |  | 0.95 (0.59~1.53) |  | 0.835 |  | | 0.94 (0.58~1.52) | |  | | 0.832 | | |  |
| Q4 | 0.99 (0.61~1.60) |  | 0.998 |  | 1.08 (0.66~1.76) |  | 0.835 |  | | 1.06 (0.65~1.72) | |  | | 0.832 | | |  |
| *P* for trend | |  | 0.998 |  |  |  | 0.835 |  | |  | |  | | 0.832 | | |  |
| **Posterior circulation stenosis** | | | | | | | | | | | | | | | | |  |
| AGR | 0.28 (0.15~0.53) |  | 0.010* |  | 0.28 (0.14~0.55) |  | 0.010* |  | | 0.27 (0.14~0.53) | |  | | 0.010* | | |  |
| AGR quartiles | | | | | | | | | | | | | | | | |  |
| Q1 | Reference |  |  |  |  |  |  |  | |  | |  | |  | | |  |
| Q2 | 0.70 (0.47~1.06) |  | 0.155 |  | 0.70 (0.46~1.06) |  | 0.120 |  | | 0.71 (0.47~1.09) | |  | | 0.192 | | |  |
| Q3 | 0.57 (0.38~0.86) |  | 0.030* |  | 0.59 (0.39~0.89) |  | 0.040* |  | | 0.60 (0.39~0.92) | |  | | 0.048* | | |  |
| Q4 | 0.59 (0.39~0.89) |  | 0.030* |  | 0.60 (0.39~0.91) |  | 0.040* |  | | 0.59 (0.38~0.90) | |  | | 0.048* | | |  |
| *P* for trend | |  | 0.030* |  |  |  | 0.040* |  | |  | |  | | 0.048* | | |  |
| **Intracranial stenosis** | | | | | | | | | | | | | | | | |  |
| AGR | 0.21 (0.10~0.43) |  | 0.010* |  | 0.20 (0.09~0.43) | | 0.010* |  | | 0.20 (0.09~0.42) | |  | | | 0.010* | |  |
| AGR quartiles | | | | | | | | | | | | | | | | |  |
| Q1 | Reference |  |  |  |  | |  | |  | |  | |  | | |  | |
| Q2 | 0.95 (0.58~1.57) |  | 0.840 |  | 0.96 (0.58~1.58) | | 0.859 | |  | | 0.93 (0.56~1.54) | |  | | | 0.774 | |
| Q3 | 0.72 (0.45~1.17) |  | 0.374 |  | 0.72 (0.44~1.17) | | 0.356 | |  | | 0.70 (0.43~1.15) | |  | | | 0.324 | |
| Q4 | 0.55 (0.34~0.88) |  | 0.040* |  | 0.55 (0.34~0.86) | | 0.047* | |  | | 0.55 (0.34~0.89) | |  | | | 0.050 | |
| *P* for trend | |  | 0.025* |  |  | | 0.035* | |  | |  | |  | | | 0.030* | |
| **Extracranial stenosis** | | | | | | | | | | | | | | | | |  |
| AGR | 0.48 (0.25~0.91) |  | 0.060 |  | 0.48 (0.25~0.94) | | 0.083 | |  | | 0.47 (0.24~0.93) | |  | | | 0.073 | |
| AGR quartiles | | | | | | | | | | | | | | | | |  |
| Q1 | Reference |  |  |  |  | |  | |  | |  | |  | | |  | |
| Q2 | 0.84 (0.55~1.27) |  | 0.574 |  | 0.84 (0.55~1.28) | | 0.685 | |  | | 0.87 (0.56~1.33) | |  | | | 0.706 | |
| Q3 | 0.82 (0.54~1.24) |  | 0.574 |  | 0.87 (0.57~1.33) | | 0.733 | |  | | 0.88 (0.57~1.36) | |  | | | 0.706 | |
| Q4 | 0.87 (0.58~1.32) |  | 0.612 |  | 0.89 (0.58~1.37) | | 0.733 | |  | | 0.88 (0.57~1.35) | |  | | | 0.706 | |
| *P* for trend | |  | 0.612 |  |  | | 0.737 | |  | |  | |  | | | 0.774 | |

Model 1: unadjusted; Model 2:adjusted for age and sex; Model 3:adjusted for age, sex, BMI, hypertension, diabetes mellitus, coronary heart disease, stroke, smoking and drinking. AGR, Albumin/Globulin Ratio; BMI, body mass index; OR, odds ratio; CI, confidence interval. **p*<0.05.

**Table S3** The association between AGR and vascular stenosis stratified by age

|  | <65 years | |  | ≥65 years | |
| --- | --- | --- | --- | --- | --- |
|  | OR (95%CI) | *p*-value |  | OR (95%CI) | *p*-value |
| **Anterior circulation stenosis** | | | | | |
| AGR (continuous) | 0.29 (0.11~0.80) | 0.017* |  | 0.79 (0.25~2.50) | 0.692 |
| AGR (quartiles) |  |  |  |  |  |
| Q1 | Reference |  |  | Reference |  |
| Q2 | 1.17 (0.56~2.43) | 0.678 |  | 0.63 (0.33~1.21) | 0.169 |
| Q3 | 0.93 (0.48~1.80) | 0.819 |  | 0.88 (0.43~1.81) | 0.720 |
| Q4 | 0.89 (0.46~1.70) | 0.718 |  | 1.20 (0.54~2.71) | 0.654 |
| **Posterior circulation stenosis** | | | | | |
| AGR (continuous) | 0.39 (0.16~0.96) | 0.041* |  | 0.17 (0.06~0.48) | 0.001** |
| AGR (quartiles) |  |  |  |  |  |
| Q1 | Reference |  |  | Reference |  |
| Q2 | 0.82 (0.44~1.55) | 0.546 |  | 0.65 (0.36~1.17) | 0.148 |
| Q3 | 0.77 (0.43~1.40) | 0.395 |  | 0.44 (0.24~0.82) | 0.010* |
| Q4 | 0.70 (0.39~1.26) | 0.234 |  | 0.52 (0.27~0.99) | 0.047* |
| **Intracranial stenosis** | | | | | |
| AGR (continuous) | 0.23 (0.08~0.66) | 0.006** |  | 0.14 (0.05~0.41) | <0.001*** |
| AGR (quartiles) |  |  |  |  |  |
| Q1 | Reference |  |  | Reference |  |
| Q2 | 1.48 (0.65~3.35) | 0.349 |  | 0.65 (0.33~1.26) | 0.198 |
| Q3 | 0.71 (0.36~1.42) | 0.333 |  | 0.79 (0.38~1.63) | 0.522 |
| Q4 | 0.77 (0.39~1.53) | 0.459 |  | 0.32 (0.16~0.65) | 0.002** |
| **Extracranial stenosis** | | | | | |
| AGR (continuous) | 0.39 (0.16~0.97) | 0.043* |  | 0.65 (0.23~1.80) | 0.404 |
| AGR (quartiles) |  |  |  |  |  |
| Q1 | Reference |  |  | Reference |  |
| Q2 | 0.85 (0.45~1.60) | 0.612 |  | 0.82 (0.46~1.47) | 0.505 |
| Q3 | 0.86 (0.47~1.56) | 0.610 |  | 0.84 (0.45~1.59) | 0.593 |
| Q4 | 0.73 (0.40~1.31) | 0.292 |  | 1.18 (0.60~2.24) | 0.628 |

Adjusted for age, sex, hypertension, diabetes mellitus; coronary heart disease, stroke, smoking and drinking. AGR, Albumin/Globulin Ratio; OR, odds ratio; CI, confidence interval. **p*<0.05, ***p*<0.001, ****p*<0.01.

**Table S4** The association between AGR and vascular stenosis stratified by sex

|  | Male | |  | Female | |
| --- | --- | --- | --- | --- | --- |
|  | OR (95%CI) | *p*-value |  | OR (95%CI) | *p*-value |
| **Anterior circulation stenosis** | | | | | |
| AGR (continuous) | 0.32 (0.12~0.89) | 0.029* |  | 0.87 (0.28~2.73) | 0.809 |
| AGR (quartiles) |  |  |  |  |  |
| Q1 | Reference |  |  | Reference |  |
| Q2 | 0.86 (0.41~1.82) | 0.701 |  | 0.85 (0.45~1.59) | 0.610 |
| Q3 | 0.75 (0.38~1.50) | 0.420 |  | 1.21 (0.60~2.45) | 0.599 |
| Q4 | 0.89 (0.45~1.76) | 0.737 |  | 1.25 (0.59~2.65) | 0.569 |
| **Posterior circulation stenosis** | | | | | |
| AGR (continuous) | 0.35 (0.14~0.88) | 0.025* |  | 0.22 (0.08~0.60) | 0.003** |
| AGR (quartiles) |  |  |  |  |  |
| Q1 | Reference |  |  | Reference |  |
| Q2 | 0.55 (0.28~1.07) | 0.079 |  | 0.84 (0.48~1.46) | 0.536 |
| Q3 | 0.61 (0.32~1.16) | 0.128 |  | 0.58 (0.32~1.05) | 0.074 |
| Q4 | 0.66 (0.35~1.23) | 0.188 |  | 0.53 (0.29~0.99) | 0.046* |
| **Intracranial stenosis** | | | | | |
| AGR (continuous) | 0.16 (0.06~0.45) | 0.001** |  | 0.22 (0.07~0.71) | 0.011* |
| AGR (quartiles) |  |  |  |  |  |
| Q1 | Reference |  |  | Reference |  |
| Q2 | 1.18 (0.53~2.62) | 0.685 |  | 0.79 (0.41~1.53) | 0.486 |
| Q3 | 0.56 (0.28~1.13) | 0.105 |  | 0.87 (0.42~1.81) | 0.714 |
| Q4 | 0.52 (0.26~1.01) | 0.055 |  | 0.55 (0.27~1.12) | 0.098 |
| **Extracranial stenosis** | | | | | |
| AGR (continuous) | 0.33 (0.13~0.86) | 0.023* |  | 0.80 (0.30~2.14) | 0.660 |
| AGR (quartiles) |  |  |  |  |  |
| Q1 | Reference |  |  | Reference |  |
| Q2 | 0.72 (0.36~1.44) | 0.353 |  | 0.96 (0.55~1.66) | 0.873 |
| Q3 | 0.83 (0.43~1.62) | 0.593 |  | 1.00 (0.56~1.79) | 1.000 |
| Q4 | 0.78 (0.41~1.48) | 0.443 |  | 0.06 (0.57~1.97) | 0.853 |

Adjusted for age, hypertension, diabetes mellitus; coronary heart disease, stroke, smoking and drinking. AGR, Albumin/Globulin Ratio; OR, odds ratio; CI, confidence interval. **p*<0.05, ***p*<0.01.
